# Enzootic and Epizootic Rabies Associated with Vampire Bats, Peru

**DOI:** 10.3201/eid1909.130083

**Published:** 2013-09

**Authors:** Rene Edgar Condori-Condori, Daniel G. Streicker, Cesar Cabezas-Sanchez, Andres Velasco-Villa

**Affiliations:** University of Georgia, Athens, Georgia, USA (R.E. Condori-Condori, D.G. Streicker);; Centers for Disease Control and Prevention, Atlanta, Georgia, USA (R.E. Condori-Condori, D.G. Streicker, A. Velasco-Villa);; Instituto Nacional de Salud Centro Nacional de Salud Pública, Lima, Peru (R.E. Condori-Condori, C. Cabezas-Sanchez)

**Keywords:** rabies, molecular epidemiology, bats, Peru, viruses, zoonoses, vampire bats

## Abstract

During the past decade, incidence of human infection with rabies virus (RABV) spread by the common vampire bat (*Desmodus rotundus*) increased considerably in South America, especially in remote areas of the Amazon rainforest, where these bats commonly feed on humans. To better understand the epizootiology of rabies associated with vampire bats, we used complete sequences of the nucleoprotein gene to infer phylogenetic relationships among 157 RABV isolates collected from humans, domestic animals, and wildlife, including bats, in Peru during 2002–2007. This analysis revealed distinct geographic structuring that indicates that RABVs spread gradually and involve different vampire bat subpopulations with different transmission cycles. Three putative new RABV lineages were found in 3 non–vampire bat species that may represent new virus reservoirs. Detection of novel RABV variants and accurate identification of reservoir hosts are critically important for the prevention and control of potential virus transmission, especially to humans.

Rabies virus (RABV; family *Rhabdoviridae*, genus *Lyssavirus*) is a bullet-shaped, single-stranded, negative-sense RNA virus with a 12-kb genome that encodes 5 structural proteins: nucleoprotein (N), phosphoprotein, matrix protein, glycoprotein, and polymerase (*1*). Over the course of its evolutionary history, RABV has established independent transmission cycles in diverse species of mesocarnivores and bats. Rabies disease remains a serious public health concern in several countries of Asia, Africa, and the Americas, where it is estimated that >50,000 fatal infections occur annually (*2*). 

In Latin America, rabies diseases are classified into 2 major epidemiologic forms, urban rabies and sylvatic rabies. For the former, dogs are the main viral reservoir host; for the latter, several species of wild carnivores and bats maintain independent rabies enzootics. Because of the widespread control of urban rabies through vaccination of domestic dogs, the common vampire bat (*Desmodus rotundus*) has emerged as the principal RABV reservoir host along the species’ natural range from Mexico to South America (*3**,**4*). The transmission and maintenance of RABV in natural populations of *D. rotundus* bats remains poorly understood, particularly within ongoing epizootics and enzootics occurring in different regions of the Americas (*5**,**6*). Active programs for the control of vampire bat–associated rabies in Latin America rely primarily on reduction of vampire bat populations by culling (*7**,**8*). Nonetheless, cross-species transmission to humans and domestic animals persists, even in areas where culling occurs regularly.

In Peru and other countries within the Amazon rainforest region, RABV transmitted by vampire bats has acquired greater epidemiologic importance because of the more frequent detection of human rabies outbreaks. This increase may reflect enhanced laboratory-based surveillance; increased awareness among public health stakeholders; or ecologic changes that promote greater contact between bats and humans, such as depletion of vampire bats’ natural prey community through hunting or habitat fragmentation. During 2002–2007, a total of 293 (77%) of the rabies cases diagnosed by the Instituto Nacional de Salud in Peru were associated with vampire bat RABV variants; the remaining 87 (23%) were attributed to RABV variants associated with dogs. In communities where vampire bats commonly feed on humans, the frequency of outbreaks depends on the transmission dynamics within the local vampire bat populations (9,10). Unfortunately, recent outbreaks in native communities of the Amazon region have been poorly characterized because of cultural constraints and local beliefs that have precluded investigators from obtaining diagnostic specimens (*11*).

Molecular epidemiology has been extensively used to determine RABV reservoir hosts in a given region or country, define the geographic distribution of the disease associated with those hosts, infer the temporal and spatial spread of the disease, identify spillover infections to nonreservoir species, describe novel RABV variants, and detect putative host shifts (*12*). The spatiotemporal epidemiology and genetic diversity of vampire bat–associated rabies in Peru have not been explored; a laboratory-based investigation conducted in 1999 addressed the comprehensive characterization of RABV in only 2 humans (*11*). Given the increasing importance of vampire bat–associated rabies in the Peruvian Amazon, comprehensive surveys of virus diversity and elucidation of geographic boundaries are needed to clarify the frequency and duration of rabies outbreaks. The goals of our study were to 1) determine the genetic diversity and geographic distribution of RABV infection associated with vampire bats; 2) clarify disease dissemination trends among affected areas; 3) detect the origins of spillover infections to other mammals; and 4) identify novel RABV lineages.

## Materials and Methods

### Virus Samples

During 2002–2007, decentralized units of the Ministry of Health of Peru collected 157 brain samples from multiple species and geographic regions of Peru (Technical Appendix Table 1). Samples were selected on the basis of identification of vampire bat or any other bat-associated rabies virus variant by using a panel of 8 monoclonal antibodies, as described (*12*). The specimens included samples from 98 cows, 26 bats, 12 humans, 9 horses, 5 goats, 2 dogs, 2 donkeys, 1 kinkajou, 1 pig, and 1 sheep. Most samples (n = 118) originated from the departments of Apurimac, Ayacucho, Cusco, Madre de Dios, and Puno, located in the southern region of the country, which is made up of inter-Andean valleys and Amazon rainforest. Twenty-six samples were from the departments of San Martin, Amazonas, Cajamarca, and Lambayeque, located in the northern region, which comprises the Andean mountains and Amazonian forests. The remaining 13 samples were from the departments of Pasco, Huanuco, and Ucayali in the central Amazon. All samples were submitted to the reference laboratory of the Instituto Nacional de Salud for RABV confirmation by fluorescent antibody testing (*13*).

### PCR and Sequencing 

Total RNA was extracted from each sample after a single passage in mouse brains by using TRIzol (Invitrogen, Carlsbad, CA, USA), according to the manufacturer’s specifications. Amplification of the complete N gene was achieved by reverse transcription PCR through 2 overlapping reactions by use of 3 published primers (Lys 001, 550F, and 304) and a modified version of primer 1066degB (*14**–**16*). The primer sets were used in the following combinations: Lys001, 5′-ACGCTTAACGAMAAA-3′; 1066degB, 5′-TCYCTGAAGAATCTTCTYTC-3′; 550F, 5′-ATGTGYGCTAAYTGGAGYAC-3′; and 304, 5′-TTGACGAAGATCTTGCTCAT-3′ (*14*–*16*). PCR products were visualized on 1.5% agarose gels, and expected size amplicons were purified by using ExoSAP-IT (USB Products Affymetrix, Inc., Cleveland, OH, USA). Cycle sequencing reactions were conducted by using Big Dye Terminator v1.1 Cycle Sequencing Kit (Applied Biosystems, Foster City, CA, USA), according to the manufacturer’s instructions. Products were analyzed on an ABI 3730 DNA analyzer (Applied Biosystems, Grand Island, NY, USA). Chromatograms were edited by using BioEdit (*17*), and sequences were assembled by using the fixed RABV SADB19 (GenBank accession no. M31046) as a template (*18*). Multiple alignments were attained by using ClustalW (*19*)

### Phylogenetic Analysis

For phylogenetic reconstructions, we retrieved complete and partial RABV sequences from GenBank that represented historical and ongoing rabies epizootics in the Americas (Technical Appendix Table 2). Other *Lyssavirus* species, such as European bat lyssavirus (EBLV) 1 (U22845) and EBLV-2 (U22846), were included as outgroups (*20*). Phylogenetic reconstructions using complete N gene sequences of the 157 isolates from Peru and 83 from GenBank were generated by using the neighbor-joining (NJ) method in MEGA 4.0 (*21*), assuming the maximum composite likelihood nucleotide substitution model. The statistical significance of branch partitions was assessed with 1,000 bootstrap replicates. We also estimated a time-scaled phylogenetic tree for the dataset comprising RABVs associated with *D. rotundus* (154 isolates from Peru and 58 from GenBank) by using BEAST version 1.7 (*22*), which uses a Bayesian coalescent framework to estimate evolutionary parameters from many possible genealogies through Markov chain Monte Carlo sampling. Our analysis used the Bayesian skyline model of population growth as a flexible demographic prior and the relaxed lognormal molecular clock to allow for rate variation among branches of the tree. Substitution models for coding positions 1+2 (CP12) and CP3 were unlinked, and substitution models in each coding position were selected by Akaike Information Criterion in jModeltest (*23*). The general time reversible model, including invariant sites and Γ distributed site heterogeneity, was applied to CP12, and time reversible model + Γ was applied to CP3. Four replicate Markov chain Monte Carlo analyses were run for 60 million generations each and combined for final estimates and construction of the maximum-clade credibility tree. Convergence across runs, appropriate burn-in periods, and effective sample sizes >200 were assessed by using Tracer (http://beast.bio.ed.ac.uk/Tracer).

## Results

### Phylogeny of RABV Isolates

**Figure 1:**
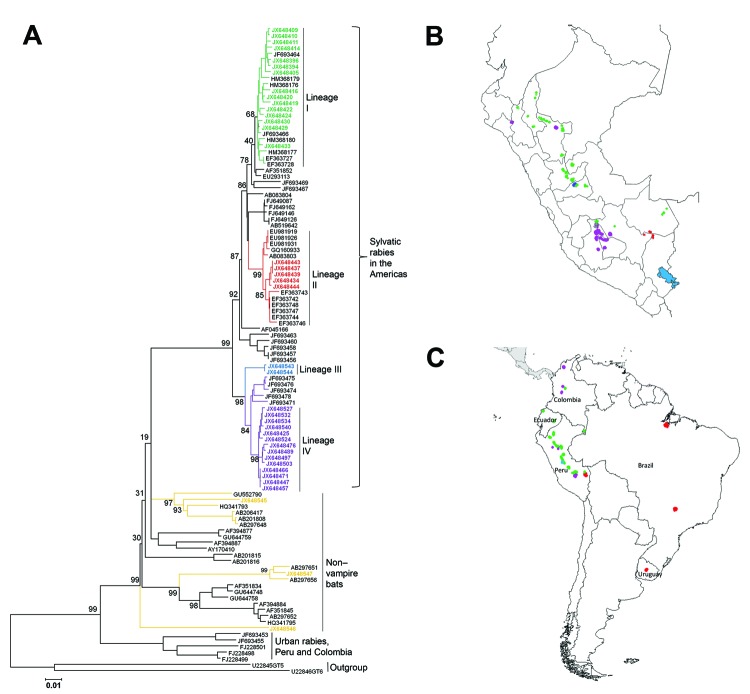
Phylogenetic and geographic comparisons of rabies virus isolates collected in Peru during 2002–2007 with representative rabies viruses circulating in South America. A) Phylogenetic tree showing relationships among virus isolates; B) locations from which viruses were isolated in Peru and South America. Colors indicate isolates from this study: green, lineage I; red, lineage II; blue, lineage III; purple, lineage IV. Gold indicates the 3 isolates collected in Peru from non–vampire bats. Additional lineage I isolates were found in Ecuador and Colombia; lineage II, Brazil and Uruguay; and lineage IV, Colombia. GenBank accession numbers are indicated. Scale bar indicates nucleotide substitutions per site.

Complete N gene sequences (1,350 nt, excluding the stop codon) were obtained from 157 specimens from humans, domesticated animals, and wildlife from 12 of the 24 departments of Peru (Technical Appendix Table 1). Pairwise similarity ranged from 85.9% to 100%, with an average pairwise identity of 97.3%. The NJ phylogenetic analysis demonstrated 2 major RABV clusters, 1 associated with *D. rotundus* bats and 1 associated with insectivorous bats. The *D. rotundus* cluster was subsequently subdivided into 4 lineages, I–IV, each with a distinctive geographic distribution within Peru; the RABVs associated with insectivorous bats segregated into 3 independent RABV lineages not previously reported in Peru (Figure 1).

Sequences within lineage I showed a widespread spatiotemporal distribution. Isolates were obtained from the departments of Amazonas, San Martin, Cajamarca, Huanuco, Ucayali, Pasco, Ayacucho, Cusco, and Madre de Dios. Inclusion of the reference sequences from GenBank revealed that lineage I had an extended spatiotemporal distribution over northern regions of South America, encompassing Ecuador and Colombia, during 1997–2007 (Figure 1) (*24*). Conversely, isolates in lineage II were detected predominately during a human rabies outbreak in the Madre de Dios and Puno departments in 2007. This lineage also grouped with an isolate from a sample found in the Cusco department in 2003 (GenBank accession no. JX648444) and with several RABV sequences reported in Brazil and Uruguay during 2004–2008 (*24*). These findings indicate that lineage II has been circulating within a larger geographic scale, perhaps reflecting virus dispersion across the Amazon region and southern South America (Figures 1). Two isolates (GenBank accession nos. JX648544 and JX648543) grouped into lineage III as an independent cluster unrelated to any previously described RABV (Figure 1). These samples were collected in 2006 from the Pozuzo district, which is located in the eastern side of the Pasco department in the central Peruvian Amazon. 

Lineage IV was the most frequently identified lineage among the isolates collected in Peru, encompassing 98 of the 157 isolates captured during 2002–2007. These results indicate this lineage’s high prevalence in cattle in the Andes. Its geographic distribution comprised the valleys of Ayacucho and Apurimac, located at 1,200–3,500 m above sea level and extended into Cusco and north into San Martin, Lambayeque, and northern Colombia. Although this lineage was predominately collected from livestock, it was also obtained from vampire bats (n = 24).

### Evolution of Vampire Bat–Associated RABV in Peru 

**Figure 2:**
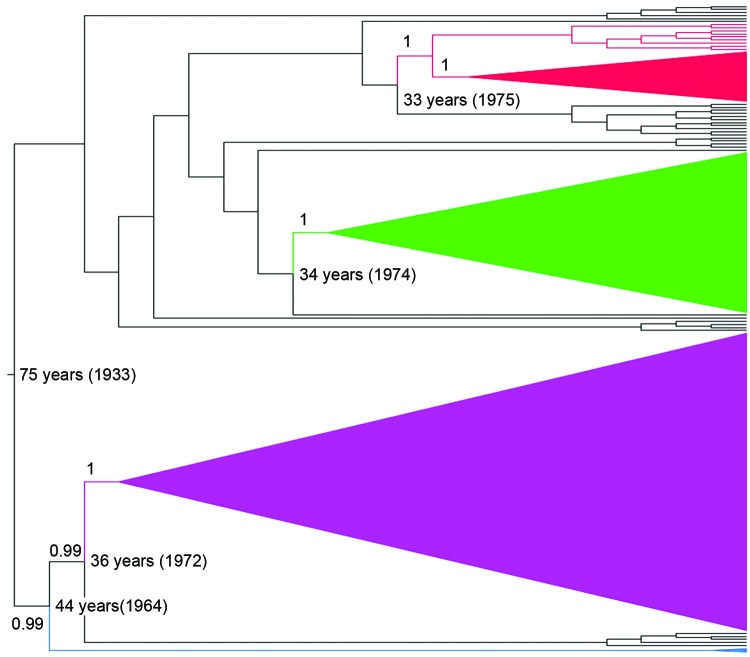
Phylogenetic tree generated by using Bayesian analysis for a 1,274-nt portion of the gene-coding sequences of rabies virus isolates collected in Peru during 2002–2007. Red, lineage II (found in Peru, Brazil, and Uruguay); green, lineage I (found in Peru, Ecuador, and Colombia); purple, lineage IV (found in Peru and Colombia); blue, lineage III (found in Peru). Posterior probabilities and lineage ages are shown for all major nodes.

By applying a Bayesian coalescent analysis to 212 serially sampled partial N sequences (1,275-bp), we inferred the time scale of RABV evolution in lineages associated with vampire bats. Consistent with previous estimates, the median rate of nucleotide substitution of vampire bat–associated RABV was 9.76 × 10^−4^ substitutions per site per year (95% highest posterior density [HPD] 6.81 × 10^−4^ to 1.3 × 10^−3^). These results would place the most recent common ancestor (MRCA) of contemporary vampire bat–associated RABVs as occurring in Peru in 1933 (95% HPD 1889–1962) (*25*). The maximum clade credibility tree (Figure 2) demonstrated similar topology to the NJ tree (Figure 1) when broader datasets were used, with vampire bat–associated RABVs differentiated into 4 phylogenetic lineages (Figure 2). As in the NJ trees, a deep division at the MRCA of vampire bat–associated RABVs separated lineages I and II from lineages III and IV (posterior probability [PP] 1.0). Major lineages appear to have been circulating for similar periods in Peru, each originating 33–44 years ago (when including the stem branch leading to current viral diversity), with extensive overlap of the 95% HPDs of the time since the MRCA for each lineage. Each RABV lineage in Peru except lineage III shared common ancestors with viruses circulating in other Latin American countries, indicating multiple viral dispersion events into or out of Peru; however, overlap of the 95% HPDs on the age of samples from Peru compared with those from other countries limited direct inference on the directionality of movement between countries. Within lineage I, samples from Ecuador and Colombia were interspersed with contemporary samples from Peru, which suggests a relatively recent spatial spread among countries. In addition, historical introductions of a similar RABV were indicated by strong posterior support (PP 0.99) for an MRCA between lineage I and samples from Colombia, Trinidad, and French Guyana in about 1973. Isolates related to lineage II were detected in Brazil and Uruguay; however, strong spatiotemporal clustering apparently separated distinct epizootics in Brazil in 2004 and Uruguay and Brazil in 2007–2008 from the human outbreak in southern Peru in 2007. A sample from a cow collected in 2003 in Peru was ancestral to samples from the 2007 human outbreak in Peru (PP 1), rather than grouping with the more contemporaneous viruses circulating in Brazil in 2004, indicating that this virus may have circulated in Peru for >4 years before the 2007 outbreak. 

As in the NJ tree, lineage III was most closely related to lineage IV (PP 0.99) but was highly divergent, sharing an MRCA in 1963 (95% HPD 1940–1979). The large genetic distance from other lineages indicates that these sporadic cases were not recently introduced from other RABV lineages circulating elsewhere in Peru but rather were part of a previously unknown vampire bat–associated rabies enzootic. No samples from other countries clustered with the lineage IV samples from Peru, suggesting that this virus has been maintained independently as a widespread rabies focus that covers the inter-Andean valleys (Ayacucho, Apurimac) and the rainforest of northern Peru (San Martin). The closest relatives of lineage IV were viruses collected in Colombia during 1994–2008, which diverged from the samples from Peru around 1972 (95% HPD 1957–1984), consistent with the enzootic maintenance of rabies over long periods within Peru.

### Novel RABV Associated with Other Wildlife in Peru

We identified 3 novel RABV variants in wildlife other than vampire bats in the southeastern region of Peru. The first variant (GenBank accession no. JX648546) was isolated from a kinkajou (*Potus flavus*) in Madre de Dios in the Amazon rainforest in southern Peru. This variant was not closely related to any previously described RABV but grouped within the larger diversity of bat-associated RABVs in the Americas (Figure 1). A second RABV variant (GenBank accession no. JX648545) was isolated from a small big-eared brown bat (*Histiotus montanus*) in Puno in southern Peru. This variant was related to RABVs found in bats of the genera *Histiotus, Nyctinomops*, and *Tadarida* from Chile and Brazil but appears to be an independent lineage; its branch length and pairwise average divergence of 5% separate it from its closest relatives. A third variant (GenBank accession no. JX648547) was found during 2008 in Paucartambo, Cusco, which is located in an inter-Andean valley at 2,900 m altitude. This sample clustered with sequences from unidentified bats from Brazil (GenBank accession nos. AB297651 and AB297656). Unfortunately, the bat from which this sample was obtained was not available for taxonomic identification.

## Discussion

Rabies epidemiology has experienced dramatic changes in Latin America during the past 4 decades because of the implementation of highly effective strategies for prevention and control of infection in dogs and the procurement of adequate postexposure prophylaxis for humans. In 2003, human rabies cases transmitted by bats outnumbered cases transmitted by dogs in Latin America (*4*), and that trend has continued. The increasing detection of RABV infection in humans in the Peruvian Amazon and the persistence of vampire bat–transmitted RABV infection in livestock highlight the need to clarify the diversity of RABV lineages circulating in Peru and the spatiotemporal dynamics of RABVs associated with vampire bats. We completed phylogenetic analysis of bat-associated RABVs collected in Peru, using samples collected from rabies-endemic areas in the Andes, during sporadic human outbreaks in the Amazon, and from previously unsurveyed wildlife host species. Our study revealed that at least 4 phylogenetic lineages of RABV are circulating in vampire bat populations in Peru; these lineages appeared to display distinctive spatiotemporal dynamics across their geographic ranges. Three of the lineages had wide geographic distributions in Peru and recent and historical relationships linked to rabies outbreaks occurring in other parts of South America (*24**,**26**–**32*). Dissemination of vampire bat–associated RABV appears to be gradual rather than involving long-distance dispersal events, as might be expected by the absence of long-distance migration and small home range of the reservoir species (*33**,**34*). Spatiotemporal analysis of lineage I, II, and IV RABVs showed that the ample distribution ranges were covered over periods no shorter than 3–4 decades. The specific movement of vampire bat–associated RABVs is difficult to assess, but the phylogenetic and evolutionary analyses we conducted indicate that lineages I and IV spread from north to south, whereas lineage II spread from south to north. Lineage III had restricted distribution in central Peru, which suggests it was part of a long-term vampire rabies enzootic that disappeared from Peru around 2006. Hence, in contrast to lineages I and IV, the local dynamics for lineage 3 were epizootic rather than enzootic. Understanding factors linked to the limited geographic distribution and apparent extinction of lineage III are important for preparing improved prevention and control practices.

Vampire bats are not a migratory species and usually inhabit places below 1,800-m altitude. Nonetheless, they may occasionally move relatively long distances and inhabit higher altitudes in response to limited food or roost availability. Movement encouraged by food supplementation may be illustrated by the distribution dynamics observed for lineage IV, which currently is mainly found along the inter-Andean valleys, an important cattle raising area in Per, which has an average altitude >2,000 m (*35*). Our data suggest that the incursion of lineage IV into the inter-Andean valleys is relatively recent (30–40 years ago) and probably occurred from northern lower lands, consistent with the likely ancestors of this lineage coming from Colombia and Ecuador (Figure 2). Because of the detrimental economic effects of vampire bat–associated rabies in the livestock industry in this region, in 2010, the government of Peru initiated intense control and prevention measures that included culling vampire bats. However, the frequency of rabies cases in livestock has been unaffected (*6*).

Our study showed that different RABV lineages may overlap temporally and geographically, which indicates that, within a rabies enzootic region, convergence or cocirculation of >1 RABV lineage may occur, perhaps in association with the maintenance of independent rabies foci by distinct vampire bat metapopulations. This observation could affect effective planning of prevention and control strategies because 1 focal point might be vulnerable to rabies reintroduction from adjacent foci, a process that could explain the persistence of the disease. Studies of population structure, gene flow, and dispersal of vampire bats within Peru and throughout the South America are necessary for corroborating observations on the dissemination dynamics of rabies associated with this species.

Although it was not the intent of this study to identify the role of rabies transmission and maintenance among species other than vampire bats, we circumstantially discovered 3 potentially novel RABV lineages in non–vampire bat hosts. This finding stresses the potential emergence of novel RABV reservoirs in the country and the need for enhanced surveillance for lyssaviruses in potential wild animal reservoirs. In Peru, the surveillance system for the detection and monitoring of human rabies cases associated with bats and other wild animals is passive; that is, cases are recorded only as they are reported. Operationally, the system is less than ideal because, even though most clinical cases of rabies in humans may be recorded, few are laboratory confirmed; consequently, the RABV variants associated with them are not typed. Rabies associated with insectivorous bats is commonly encountered in countries such as the United States, where 1 or 2 cases of rabies occur in humans each year (*36*). A bat rabies surveillance system such as the one in place in the United States, which tests >20,000 bats and confirms ≈1,400 infections each year, relies heavily on submissions of sick or dead bats to rabies diagnostic facilities from the general public (*36*). This public participation in the process has been augmented by active educational programs that emphasize the potential risk for rabies transmission from bats to humans, pets, and livestock. Human rabies associated with insectivorous bats has been reported in other countries in Latin America, such as Chile and Mexico (*37**,**38*), but the role of these bats in rabies transmission to humans is largely unknown in Peru. Therefore, better understanding of these transmission cycles and better programs for the taxonomic identification of bats with rabies should be implemented.

We also identified RABV in a kinkajou; this strain that was not closely related to any known RABV. We could not determine whether this animal represented a single spillover infection from a previously unknown bat reservoir or an emerging host shift with ongoing transmission within kinkajous. Kinkajous are in the same taxonomic family (*Procyonidae*) as raccoons (*Procyon lotor*) (*39*), which are a well-established rabies reservoir in North America. This relationship suggests that kinkajou could serve as an emerging RABV reservoir if the traits that enable the establishment of RABV reservoirs are conserved along the phylogeny of procyonids. Serologic surveys and enhanced surveillance would be useful for further exploring this possibility.

In conclusion, our study demonstrates the presence of diverse RABV lineages associated with vampire bats and several other species in Peru. Although our research was limited by the restrictions of passive surveillance data, RABV lineages in vampire bats appear to show distinct spatiotemporal patterns, with 2 lineages that were abundant and widely distributed throughout the study period and 2 others that occurred more sporadically, consistent with enzootic and epizootic dynamics. Further discrimination of transmission cycles and their drivers will be crucial for prediction of the frequency of outbreaks in humans and domestic animals and, ultimately, for the design of informed strategies for rabies control in this region.

Technical Appendix Isolates of rabies virus from Peru and representative strains from the Americas included in study of rabies virus in Peru.
