# Supplementary material for: Enzootic and Epizootic Rabies Associated with Vampire Bats, Peru
Source: Emerg Infect Dis. 2013 Sep;19(9):1463–9. doi: 10.3201/eid1909.130083 (PMC3810916; doi:10.3201/eid1909.130083)
Supplement: Technical Appendix — Isolates of rabies virus from Peru and representative strains from the Americas included in study of rabies virus in Peru. [file 13-0083-Techapp-s1.pdf]

Publisher: CDC; Journal: Emerging Infectious Diseases
Article Type: Research; Volume: 19; Issue: 9; Year: 2013; Article ID: 13-0083
DOI: 10.3201/eid1809.130083; TOC Head: Research
Article DOI: http://dx.doi.org/10.3201/eid1909.130083

Enzootic and Epizootic Rabies Associated with Vampire Bats, Peru

Technical Appendix

Technical Appendix Table 1. Isolates of rabies virus from Peru included in this study*

No,	Identification no. and genetic cluster	Animal source	Department	Province	District	Year	GenBank no.	Lineage
1	PERAPUBV1539_02	Bovine	Apurimac	Andahuaylas	Pacucha	2002	JX648538	IV
2	PERAYABV2138_02	Bovine	Ayacucho	La Mar		2002	JX648457	IV
3	PERAPUBV3040_02	Bovine	Apurimac	Andahuaylas	Pacucha	2002	JX648540	IV
4	PERAPUHR3981_02	Horse	Apurimac	Andahuaylas	Andarapa	2002	JX648525	IV
5	PERAPUBV3982_02	Bovine	Apurimac	Andahuaylas	Pacucha	2002	JX648541	IV
6	PERAPUBV5119_02	Bovine	Apurimac	Andahuaylas	Pacucha	2002	JX648539	IV
7	PERAYABV10574_02	Bovine	Ayacucho	La Mar		2002	JX648460	IV
8	PERAYAGT10576_02	Goat	Ayacucho	La Mar	Chilcas	2002	JX648471	IV
9	PERAYAGT10578_02	Goat	Ayacucho	Huamanga		2002	JX648497	IV
10	PERAPUBT12725_02	Bat	Apurimac	Chincheros	Ongoy	2002	JX648518	IV
11	PERHUABV14643_02	Bovine	Huanuco	Puerto Inca	Codo de Pozuzo	2002	JX648402	I
12	PERSMADG15667_02	Dog	San Martin	Rioja	Awajun	2002	JX648426	I
13	PERAYABV17556_02	Bovine	Ayacucho	La Mar	Chilcas	2002	JX648465	IV
14	PERSMABV18452_02	Bovine	San Martin	Rioja	Elias Soplin	2002	JX648427	I
15	PERAYABV19596_02	Bovine	Ayacucho	La Mar	San Miguel	2002	JX648458	IV
16	PERAYABV24974_02	Bovine	Ayacucho	La Mar	San Miguel	2002	JX648456	IV
17	PERAPUBV25686_02	Bovine	Apurimac	Chincheros	Ongoy	2002	JX648522	IV
18	PERAPUBV25773_02	Bovine	Apurimac	Abancay	Huanipaca	2002	JX648528	IV
19	PERAPUBV25775_02	Bovine	Apurimac	Abancay	Huanipaca	2002	JX648529	IV
20	PERSMAHR27577_02	Horse	San Martin	Lamas	Lamas	2002	JX648532	IV
21	PERAPUBV27641_02	Bovine	Apurimac	Chincheros	Huaccana	2002	JX648498	IV
22	PERAYAHR27817_02	Horse	Ayacucho	Huamanga	Ocros	2002	JX648505	IV
23	PERAYABV27821_02	Bovine	Ayacucho	La Mar	San Miguel	2002	JX648472	IV
24	PERAYABV29517_02	Bovine	Ayacucho	La Mar	Anco	2002	JX648468	IV
25	PERAYABV32068_02	Bovine	Ayacucho	La Mar	san Miguel	2002	JX648475	IV
26	PERAPUBT32653_02	Bat	Apurimac	Abancay	Huanipaca	2002	JX648533	IV
27	PERAPUBT32717_02	Bat	Apurimac	Abancay	Huanipaca	2002	JX648537	IV
28	PERSMABV33299_02	Bovine	San Martin	San Martin	Tarapoto	2002	JX648412	I
29	PERSMABV33842_02	Bovine	San Martin	San Martin	Tarapoto	2002	JX648413	I
30	PERSMAHR35351_02	Horse	San Martin	Lamas	Lamas	2002	JX648424	I
31	PERAMABV36425_02	Bovine	Amazonas	Utcubamba	Cajaruro	2002	JX648400	I
32	PERAYABV39012_02	Bovine	Ayacucho	La Mar	San Miguel	2002	JX648463	IV
33	PERAPUBV41363_02	Bovine	Apurimac	Chincheros	Huaccana	2002	JX648501	IV
34	PERAPUBV197_03	Bovine	Apurimac	Chincheros	Huaccana	2003	JX648507	IV
35	PERAPUBV249_03	Bovine	Apurimac	Chincheros	Huaccana	2003	JX648508	IV
36	PERAPUBV250_03	Bovine	Apurimac	Chincheros	Huaccana	2003	JX648500	IV
37	PERAYABV1234_03	Bovine	Ayacucho	La Mar	San Miguel	2003	JX648467	IV
38	PERSMAHR6074_03	Horse	San Martin	San Martin	Chazuta	2003	JX648419	I
39	PERAYABV12387_03	Bovine	Ayacucho	La Mar	Anco	2003	JX648474	IV
40	PERAYAHR17815_03	Horse	Ayacucho	La Mar	San Miguel	2003	JX648466	IV
41	PERUCAHM23580_03	Human	Ucayali	Padre Abad	Irazola	2003	JX648418	I
42	PERAYAGT24135_03	Goat	Ayacucho	La Mar	San Miguel	2003	JX648531	IV
43	PERAYABV24139_03	Bovine	Ayacucho	Huamanga	Ocros	2003	JX648536	IV
44	PERUCAHM24278_03	Human	Ucayali	Padre Abad	Padre Abad	2003	JX648416	I
45	PERAYABT26736_03	Bat	Ayacucho	La Mar	San Miguel	2003	JX648530	IV
46	PERAYASH26745_03	Sheep	Ayacucho	Huamanga	Ocros	2003	JX648504	IV
47	PERAYABV29265_03	Bovine	Ayacucho	La Mar	Chungui	2003	JX648535	IV
48	PERCUSBV38910_03	Bovine	Cusco	Paucartambo	Kosñipata	2003	JX648444	II
49	PERHUABV39200_03	Bovine	Huanuco	Leoncio Prado	José Crespo y	2003	JX648414	I

No,	Identification no. and genetic cluster	Animal source	Department	Province	District	Year	GenBank no.	Lineage
					Castillo			
50	PERHUABV41340_03	Bovine	Huanuco	Leoncio Prado	José Crespo y Castillo	2003	JX648415	I
51	PERPASBV43903_03	Bovine	Pasco	Oxapampa	Pozuzo	2003	JX648417	I
52	PERAYABV10421_04	Bovine	Ayacucho	Huamanga	Ocros	2004	JX648502	IV
53	PERAYABV11462_04	Bovine	Ayacucho	Huamanga	Ocros	2004	JX648503	IV
54	PERAYABT13771_04	Bat	Ayacucho	La Mar	Tambo	2004	JX648459	IV
55	PERSMABV14677_04	Bovine	San Martin	Tocache	Tocache	2004	JX648401	I
56	PERLAMDG16670_04	Dog	Lambayeque	Ferreñafe	Incahuasi	2004	JX648489	IV
57	PERAYABV17034_04	Bovine	Ayacucho	Vilcashuaman	Concepcion	2004	JX648495	IV
58	PERSMAHR17048_04	Horse	San Martin	Rioja	Nueva Cajamarca	2004	JX648429	I
59	PERSMAHR17053_04	Horse	San Martin	Rioja	Nueva Cajamarca	2004	JX648428	I
60	PERAYABV24247_04	Bovine	Ayacucho	La Mar	Chilcas	2004	JX648506	IV
61	PERAYABT24249_04	Bat	Ayacucho	Huamanga	Ocros	2004	JX648499	IV
62	PERCUSHR24845_04	Horse	Cusco	La Convencion	Vilcabamba	2004	JX648534	IV
63	PERCUSBV26090-04	Bovine	Cusco	La Convencion	Echarate	2004	JX648393	I
64	PERMDIBV30578_04	Bovine	Madre de Dios	Tambopata	Laberinto	2004	JX648404	I
65	PERAPUBV43925_04	Bovine	Apurimac	Chincheros	Ongoy	2004	JX648447	IV
66	PERSMABV54395_04	Bovine	San Martin	Picota	Picota	2004	JX648431	I
67	PERAMAPG2034-05	Pig	Amazonas	Condorcanqui	Rio Santiago	2005	JX648408	I
68	PERAMABV2039_05	Bovine	Amazonas	Condorcanqui	Rio Santiago	2005	JX648409	I
69	PERAMABV2040_05	Bovine	Amazonas	Condorcanqui	Rio Santiago	2005	JX648406	I
70	PERAPUBV5736_05	Bovine	Apurimac	Andahuaylas	Pacobamba	2005	JX648527	IV
71	PERAPUBV5737_05	Bovine	Apurimac	Andahuaylas	Pacobamba	2005	JX648526	IV
72	PERAYABV14916_05	Bovine	Ayacucho	Huamanga	Ocros	2005	JX648448	IV
73	PERPASBV25193_05	Bovine	Pasco	Oxapampa	Puerto Bermudez	2005	JX648394	I
74	PERPASBV27761_05	Bovine	Pasco	Oxapampa	Puerto Bermudez	2005	JX648395	I
75	PERMDIBV29509_05	Bovine	Madre de Dios	Tambopata	Las Piedras	2005	JX648405	I
76	PERAYABV35266-05	Bovine	Ayacucho	Huamanga	Acoria	2005	JX648450	IV
77	PERAMABV37027_05	Bovine	Amazonas	Condorcanqui	Nieva	2005	JX648403	I
78	PERAMABV41508_05	Bovine	Amazonas	Condorcanqui	Nieva	2005	JX648420	I
79	PERAYADK47065_05	Donkey	Ayacucho	La Mar	Anco	2005	JX648542	IV
80	PERAYADK50031_05	Donkey	Ayacucho	Huanta	Sivia	2005	JX648422	I
81	PERSMABV1897_06	Bovine	San Martin	El Dorado		2006	JX648399	I
82	PERSMABV1898_06	Bovine	San Martin	El Dorado		2006	JX648398	I
83	PERAPUBT4220_06	Bat	Apurimac	Chincheros	Huaccana	2006	JX648482	IV
84	PERAPUBT4221_06	Bat	Apurimac	Chincheros	Huaccana	2006	JX648492	IV
85	PERAMABV4953_06	Bovine	Amazonas	Condorcanqui	Rio Santiago	2006	JX648407	I
86	PERPASBV5714_06	Bovine	Pasco	Oxapampa	Pozuzo	2006	JX648544	III
87	PERAPUBT7039_06	Bat	Apurimac	Chincheros	Huaccana	2006	JX648483	IV
88	PERAPUBT7043_06	Bat	Apurimac	Chincheros	Huaccana	2006	JX648493	IV
89	PERAPUBT9773_06	Bat	Apurimac	Andahuaylas	Talavera	2006	JX648520	IV
90	PERAPUBT9776_06	Bat	Apurimac	Andahuaylas	Talavera	2006	JX648523	IV
91	PERAPUBT10342_06	Bat	Apurimac	Chincheros	Ongoy	2006	JX648524	IV
92	PERAPUBV12463_06	Bovine	Apurimac	Chincheros	Chincheros	2006	JX648484	IV
93	PERPASBV14803_06	Bovine	Pasco	Oxapampa	Pozuzo	2006	JX648543	III
94	PERAPUBV16406_06	Bovine	Apurimac	Chincheros	Chincheros	2006	JX648494	IV
95	PERAPUBT18510_06	Bat	Apurimac	Chincheros	Chincheros	2006	JX648480	IV
96	PERAPUBV20237_06	Bovine	Apurimac	Chincheros	Chincheros	2006	JX648491	IV
97	PERAPUBV24266_06	Bovine	Apurimac	Andahuaylas	Andarapa	2006	JX648521	IV
98	PERAPUBV28489_06	Bovine	Apurimac	Chincheros	Cocharcas	2006	JX648490	IV
99	PERAYABV28771_06	Bovine	Ayacucho	La Mar	Anco	2006	JX648453	IV
100	PERAYABV30524_06	Bovine	Ayacucho	Huamanga	Ocros	2006	JX648461	IV
101	PERAYABV31905_06	Bovine	Ayacucho	La Mar	Anco	2006	JX648452	IV
102	PERAYABV32674_06	Bovine	Ayacucho	La Mar	San Miguel	2006	JX648462	IV
103	PERAPUBV35975_06	Bovine	Apurimac	Chincheros	Anco Huallo	2006	JX648496	IV
104	PERAPUBT36009_06	Bat	Apurimac	Chincheros	Anco Huallo	2006	JX648481	IV
105	PERAYABT37810_06	Bat	Ayacucho	La Mar	San Miguel	2006	JX648470	IV
106	PERAYABV37818_06	Bovine	Ayacucho	La Mar	San Miguel	2006	JX648473	IV
107	PERAPUBV40999_06	Bovine	Apurimac	Andahuaylas	Andarapa	2006	JX648519	IV
108	PERAPUBT42668_06	Bat	Apurimac	Andahuaylas	Andahuaylas	2006	JX648510	IV
109	PERAPUBT42684_06	Bat	Apurimac	Andahuaylas	Andahuaylas	2006	JX648515	IV
110	PERAYAGT42935_06	Goat	Ayacucho	La Mar	San Miguel	2006	JX648469	IV
111	PERAPUBT44948_06	Bat	Apurimac	Andahuaylas	Andahuaylas	2006	JX648512	IV

Publisher: CDC; Journal: Emerging Infectious Diseases
Article Type: Research; Volume: 19; Issue: 9; Year: 2013; Article ID: 13-0083
DOI: 10.3201/eid1809.130083; TOC Head: Research

No,	Identification no. and genetic cluster	Animal source	Department	Province	District	Year	GenBank no.	Lineage
112	PERAPUBV45653_06	Bovine	Apurimac	Andahuaylas	Kishuara	2006	JX648514	IV
113	PERAPUBT46404_06	Bat	Apurimac	Chincheros	Huaccana	2006	JX648488	IV
114	PERAMABV51713_06	Bovine	Amazonas	Luya	Ocalli	2006	JX648430	I
115	PERMDIHM1710_07	Human	Madre de Dios	Tambopata	Inambari	2007	JX648434	II
116	PERMDIKN1766_07	*Potus flavus*	Madre de Dios	Tambopata	Las Piedras	2007	JX648546	NA
117	PERMDIHM2987_07	Human	Madre de Dios	Tambopata	Inambari	2007	JX648435	II
118	PERAYABV5591_07	Bovine	Ayacucho	Vilcashuaman	Vilcashuaman	2007	JX648487	IV
119	PERAYAGT5592_07	Goat	Ayacucho	Vilcashuaman	Vilcashuaman	2007	JX648486	IV
120	PERMDIHM5710_07	Human	Madre de Dios	Tambopata	Inambari	2007	JX648437	II
121	PERMDIHM5711_07	Human	Madre de Dios	Tambopata	Inambari	2007	JX648436	II
122	PERPUNHM7172_07	Human	Puno	Carabaya	Ayapata	2007	JX648441	II
123	PERAPUBV7745_07	Human	Cusco	Quispicanchi	Marcapata	2007	JX648442	II
124	PERCUSHM8217_07	Human	Cusco	Quispicanchi	Marcapata	2007	JX648440	II
125	PERSMABV8429_07	Bovine	San Martin	Tocache	Shunte	2007	JX648423	I
126	PERCUSHM8714-07	Human	Cusco	Quispicanchi	Marcapata	2007	JX648443	II
127	PERPUNHM9090_07	Human	Puno	Carabaya	San Gaban	2007	JX648439	II
128	PERCUSHM9449_07	Human	Cusco	Cusco	San Sebastian	2007	JX648438	II
129	PERAYABV9870_07	Bovine	Ayacucho	Huamanga	Ocros	2007	JX648449	IV
130	PERAPUBV10320_07	Bovine	Apurimac	Chincheros	Cocharcas	2007	JX648479	IV
131	PERAPUBT10370_07	Bat	Apurimac	Andahuaylas	Pacucha	2007	JX648516	IV
132	PERSMABV10590-07	Bovine	San Martin	Moyobamba	Moyobamba	2007	JX648432	I
133	PERAPUBV10742_07	Bovine	Apurimac	Chincheros	Cocharcas	2007	JX648485	IV
134	PERCUSBV10999_07	Bovine	Cusco	La Convencion	Kimbiri	2007	JX648396	I
135	PERCUSBV11000_07	Bovine	Cusco	La Convencion	Kimbiri	2007	JX648392	I
136	PERAPUBT11043_07	Bat	Apurimac	Chincheros	Cocharcas	2007	JX648478	IV
137	PERAPUBT11045_07	Bat	Apurimac	Chincheros	Chincheros	2007	JX648477	IV
138	PERAYABV11313_07	Bovine	Ayacucho	La Mar	San Miguel	2007	JX648411	I
139	PERAYABV11314_07	Bovine	Ayacucho	Huamanga	Ocros	2007	JX648464	IV
140	PERAYABV11316_07	Bovine	Ayacucho	La Mar	San Miguel	2007	JX648455	IV
141	PERAPUBT12448_07	Bat	Apurimac	Andahuaylas	Andarapa	2007	JX648517	IV
142	PERAPUBV12450_07	Bovine	Apurimac	Andahuaylas	Andarapa	2007	JX648511	IV
143	PERAYABV12690_07	Bovine	Ayacucho	La Mar	Luis Carranza	2007	JX648445	IV
144	PERHUABV13139_07	Bovine	Huanuco	Leoncio Prado	Hermilio Valdizan	2007	JX648410	I
145	PERAPUBV13361_07	Bovine	Apurimac	Chincheros	Huaccana	2007	JX648476	IV
146	PERAYABV13485_07	Bovine	Ayacucho	La Mar	San Miguel	2007	JX648446	IV
147	PERPUNBT13526_07	*Histiotus montanus*	Puno	Carabaya	Ayapata	2007	JX648545	NA
148	PERCAJBV13590_07	Bovine	Cajamarca	San Ignacio	San Ignacio	2007	JX648433	I
149	PERAPUBV13695_07	Bovine	Apurimac	Andahuaylas	Kishuara	2007	JX648509	IV
150	PERHUABV13735-07	Bovine	Huanuco	Leoncio Prado	Rupa Rupa	2007	JX648425	I
151	PERPASBV13969-07	Bovine	Pasco	Oxapampa	Puerto Bermudez	2007	JX648421	I
152	PERAYABV14465_07	Bovine	Ayacucho	La Mar	Chungui	2007	JX648454	IV
153	PERAPUBV15558_07	Bovine	Apurimac	Andahuaylas	Kishuara	2007	JX648513	IV
154	PERCUSBV15589-07	Bovine	Cusco	La Convencion	Kimbiri	2007	JX648391	I
155	PERAYABV1200456_07	Bovine	Ayacucho	La Mar	Luis Carranza	2007	JX648451	IV
156	PERSMABV1209567_07	Bovine	San Martin	Moyobamba	Moyobamba	2007	JX648397	I
157	PERCUSBT0812565_08	Non–vampire bat	Cusco	Paucartambo	Paucartambo	2008	JX648547	NA

*NA, not applicable.

Technical Appendix Table 2. Representative strains from the Americas included in this study

No.	Identification no. and genetic cluster	Animal source	Country	Year	GenBank no.	Reference
1	H02/08	Human	Colombia	2008	JF693457	Velasco-Villa et al. (unpub. data)
2	H01/08	Human	Colombia	2008	JF693456	Velasco-Villa et al. (unpub. data)
3	CT1/06	Cat	Colombia	2006	JF693458	Velasco-Villa et al. (unpub. data)
4	H04/08	Human	Colombia	2008	JF693460	Velasco-Villa et al. (unpub. data)
5	BV6/97	Cattle	Colombia	1997	JF693463	Velasco-Villa et al. (unpub. data)
6	BRhr31	Horse	Brazil	1998	AB083804	Ito et al. (*1*)
7	BRbv30	Cattle	Brazil	1999	AB083803	Ito et al. (*1*)
8	8MariliaSP1700B	Cattle	Brazil	2008	GQ160938	Macedo et al. (*2*)
9	IP6770U/08	Cattle	Uruguay	2008	EU981919	Guarino et al. (unpub. data)
10	IP6775U/08	Equine	Uruguay	2008	EU981923	Guarino et al. (unpub. data)
11	IP6781U/08	Cattle	Uruguay	2008	EU981929	Guarino et al. (unpub. data)
12	IP6778U/08	Cattle	Uruguay	2008	EU981926	Guarino et al. (unpub. data)
13	IP6785U/07	*D. rotundus*	Uruguay	2007	EU981931	Guarino et al. (unpub. data)
14	08IacriSP3577B	Cattle	Brazil	2008	GQ160933	Macedo et al. (*2*)
15	08IacriSP4001B	Cattle	Brazil	2008	GQ160934	Macedo et al. (*2*)
16	IP3066P/04H	Human	Brazil	2004	EF363742	Castilho et al. (*3*)
17	IP3072P/0H4	Human	Brazil	2004	EF363747	Castilho et al. (*3*)
18	IP3068P/0H4	Human	Brazil	2004	EF363744	Castilho et al. (*3*)
19	IP3522P/04H	Human	Brazil	2004	EF363748	Castilho et al. (*3*)
20	IP3067Portel_Para	Human	Brazil	2004	EF363743	Castilho et al. (*3*)
21	IP3070P/04H	Human	Brazil	2004	EF363746	Castilho et al. (*3*)
22	IP2617	Cattle	Brazil	2000	FJ649126	Carnieli et al. (*4*)
23	BR-DR1	*D. rotundus*	Brazil	2000	AB519642	Mochizuki et al. (*5*)
24	IP7518	Cattle	Brazil	2001	FJ649170	Carnieli et al. (*4*)
25	IP3602	Cattle	Brazil	1999	FJ649087	Carnieli et al. (*4*)
26	IP1959	Cattle	Brazil	2000	FJ649125	Carnieli et al. (*4*)
27	IP5347	Cattle	Brazil	2000	FJ649146	Carnieli et al. (*4*)
28	IP5458	Cattle	Brazil	2001	FJ649168	Carnieli et al. (*4*)
29	IP6261	Cattle	Brazil	2000	FJ649152	Carnieli et al. (*4*)
30	brdrusp100/07	*D. rotundus*	Brazil	2007	GU592648	Campos et al. (*6*)
31	IP1501	Cattle	Brazil	2001	FJ649167	Carnieli et al. (*4*)
32	IP1064	Cattle	Brazil	2001	FJ649162	Carnieli et al. (*4*)
33	HR1/02	Horse	Colombia	2002	JF693469	Velasco-Villa et al. (unpub. data)
34	H02/07	Human	Colombia	2007	JF693470	Velasco-Villa et al. (unpub. data)
35	BV1/03	Cattle	Colombia	2003	JF693468	Velasco-Villa et al. (unpub. data)
36	BV7/97	Cattle	Colombia	1997	JF693467	Velasco-Villa et al. (unpub. data)
37	DR.Td2; V325	Cattle	Trinidad Tobago	1995	AF351852	Nadin-Davis et al. (*7*)
38	IP4405EQ/07BOV	Cattle	Ecuador	2007	HM368176	Castilho et al. (*3*)
39	IP4404EQ/07BOV	Cattle	Ecuador	2007	HM368180	Castilho et al. (*3*)
40	IP4406EQ/07BOV	Cattle	Ecuador	2007	HM368177	Castilho et al. (*3*)
41	IP4407EQ/07BOV	Cattle	Ecuador	2007	HM368178	Castilho et al. (*3*)
42	IP2293EQ/05H	Human	Ecuador	2005	EF363727	Castilho et al. (*3*)
43	IP2294EQ/05H	Human	Ecuador	2005	EF363728	Castilho et al. (*3*)
44	BV1/06	Cattle	Colombia	2006	JF693466	Velasco-Villa et al. (unpub. data)
45	IP4403EQ/07BOV	Cattle	Ecuador	2007	HM368179	Castilho et al. (*3*)
46	BV8/97	Cattle	Colombia	1997	JF693465	Velasco-Villa et al. (unpub. data)
47	CT1/97	Cat	Colombia	1997	JF693464	Velasco-Villa et al. (unpub. data)

No.	Identification no. and genetic cluster	Animal source	Country	Year	GenBank no.	Reference
48	9001FRA	Dog	French Guyana	1990	EU293113	Delmas et al. (*8*)
49	pehm3230	Human	Peru	1996	AF045166	Warner et al. (*9*)
50	484	Cattle	Argentina	1996	KC758860	Included in this study
51	109	Cattle	Argentina	2001	KC758861	Included in this study
52	795	Cattle	Argentina	2007	KC758862	Included in this study
53	02	Cattle	Argentina	1999	KC758863	Included in this study
54	CT2/06	Cat	Colombia	2006	JF693474	Velasco-Villa et al. (unpub. data)
55	H02/94	Human	Colombia	1994	JF693476	Velasco-Villa et al. (unpub. data)
56	H04/95	Human	Colombia	1995	JF693475	Velasco-Villa et al. (unpub. data)
57	H03/08	Human	Colombia	2008	JF693478	Velasco-Villa et al. (unpub. data)
58	CT1/94	Cat	Colombia	1994	JF693471	Velasco-Villa et al. (unpub. data)
59	IP1992/05	*Histiotus velatus*	Brazil	2005	GU552790	Oliveira et al. (*10*)
60	Hm860	*H. macrotus*	Chile	2007	HQ341793	Yung et al. (unpub. data)
61	BR-Pbt4	*Molossus molossus*	Brazil	2003	AB206417	Shoji et al. (*11*)
62	BR-NL3	*Nyctinomops laticaudatus*	Brazil	2001	AB201808	Kobayashi et al. (*12*)
63	BR-TL1	*Tadarida laticaudata*	Brazil	1990	AB297648	Kobayashi et al. (*13*)
64	905	*Plecotus townsendii*	USA	1989	AF394877	Rohde et al. (*14*)
65	CA178	*Corynorhinus townsendii*	USA	2003	GU644759	Streicker et al. (*15*)
66	804	*Eptesicus fuscus*	USA	1987	AF394887	Rohde et al. (*14*)
67	5441	Skunk	USA	2001	AY170410	Leslie et al. (*16*)
68	BR-MM1	*M. molossus*	Brazil	1999	AB201815	Kobayashi et al. (*12*)
69	BR-MM2	*M. molossus*	Brazil	2002	AB201816	Kobayashi et al. (*12*)
70	BR-BAT13	Non–vampire bat	Brazil	2003	AB297651	Kobayashi et al. (*13*)
71	BR-BAT27	Non–vampire bat	Brazil	2006	AB297656	Kobayashi et al. (*13*)
72	SHCAN	*Lasionycteris noctivagans*	Canada	1992	AF351834	Nadin-Davis et al. (*7*)
73	MI1100	*Myotis lucifugus*	USA	2005	GU644748	Streicker et al. (*15*)
74	TX5168	*Perimyotis subflavus*	USA	2004	GU644758	Streicker et al. (*15*)
75	446	*Lasiurus cinereus*	USA	1982	AF394884	Rohde et al. (*14*)
76	LC1- 92RABL2108	*L. cinereus*	Canada	1992	AF351845	Nadin-Davis et al. (*7*)
77	BR-BAT15	Non-Vampire	Brazil	2003	AB297652	Kobayashi et al. (*13*)
78	Lc1564	*L. cinereus*	Chile	2009	HQ341795	Yung et al. (unpub. data)
79	C14/95	Dog	Colombia	1995	JF693453	Velasco-Villa et al. (unpub. data)
80	H01/00	Human	Colombia	2000	JF693455	Velasco-Villa et al. (unpub. data)
81	4355Perudg2004	Dog	Peru	2004	FJ228498	Velasco-Villa et al. (unpub. data)
82	26200PeruFx2004	Fox	Peru	2004	FJ228501	Velasco-Villa et al. (unpub. data)
83	4352Perudg2004	Dog	Peru	2004	FJ228499	Velasco-Villa et al. (unpub. data)

References

1. Ito M, Itou T, Shoji Y, Sakai T, Ito FH, Arai YT, et al. Discrimination between dog-related and vampire bat–related rabies viruses in Brazil by strain-specific reverse transcriptase-polymerase chain reaction and restriction fragment length polymorphism analysis. J Clin Virol. 2003;26:317–30. PubMed http://dx.doi.org/10.1016/S1386-6532(02)00048-3

2. Macedo CI, Carnieli Junior P, Fahl Wde O, Lima JY, Oliveira Rde N, Achkar SM, et al. Genetic characterization of rabies virus isolated from bovines and equines between 2007 and 2008, in the

States of São Paulo and Minas Gerais. Rev Soc Bras Med Trop. 2010;43:116–20. PubMed http://dx.doi.org/10.1590/S0037-86822010000200002

3. Castilho JG, Carnieli P Jr, Durymanova EA, Fahl Wde O, Oliveira Rde N, Macedo CI, et al. Human rabies transmitted by vampire bats: antigenic and genetic characterization of rabies virus isolates from the Amazon region (Brazil and Ecuador). Virus Res. 2010;153:100–5. PubMed http://dx.doi.org/10.1016/j.virusres.2010.07.012

4. Carnieli P Jr, Castilho JG, Fahl Wde O, Veras NM, Timenetsky Mdo C. Genetic characterization of Rabies virus isolated from cattle between 1997 and 2002 in an epizootic area in the state of São Paulo, Brazil. Virus Res. 2009;144:215–24. PubMed http://dx.doi.org/10.1016/j.virusres.2009.05.001

5. Mochizuki N, Kobayashi Y, Sato G, Hirano S, Itou T, Ito FH, et al. Determination and molecular analysis of the complete genome sequence of two wild-type rabies viruses isolated from a haematophagous bat and a frugivorous bat in Brazil. J Vet Med Sci. 2011;73:759–66. PubMed http://dx.doi.org/10.1292/jvms.10-0238

6. Campos AC, Melo FL, Romano CM, Araujo DB, Cunha EM, Sacramento DR, et al. One-step protocol for amplification of near full-length cDNA of the rabies virus genome. J Virol Methods. 2011;174:1–6. PubMed http://dx.doi.org/10.1016/j.jviromet.2011.03.030

7. Nadin-Davis SA, Huang W, Armstrong J, Casey GA, Bahloul C, Tordo N, et al. Antigenic and genetic divergence of rabies viruses from bat species indigenous to Canada. Virus Res. 2001;74:139–56. PubMed http://dx.doi.org/10.1016/S0168-1702(00)00259-8

8. Delmas O, Holmes EC, Talbi C, Larrous F, Dacheux L, Bouchier C, et al. Genomic diversity and evolution of the lyssaviruses. PLoS ONE. 2008;3:e2057. PubMed http://dx.doi.org/10.1371/journal.pone.0002057

9. Warner CK, Zaki SR, Shieh W-J, Whitfield SG, Smith JS, Orciari LA, et al. Laboratory investigation of human deaths from vampire bat Rabies in Peru. Am J Trop Med Hyg. 1999;60:502–7. PubMed

10. Oliveira RN, de Souza SP, Lobo RS, Castilho JG, Macedo CI, Carnieli P Jr, et al. Rabies virus in insectivorous bats: implications of the diversity of the nucleoprotein and glycoprotein genes for molecular epidemiology. Virology. 2010;405:352–60. PubMed http://dx.doi.org/10.1016/j.virol.2010.05.030

11. Shoji Y, Kobayashi Y, Sato G, Gomes AA, Itou T, Ito FH, et al. Genetic and phylogenetic characterization of rabies virus isolates from wildlife and livestock in Paraiba, Brazil. Acta Virol. 2006;50:33–7. PubMed

12. Kobayashi Y, Sato G, Shoji Y, Sato T, Itou T, Cunha EM, et al. Molecular epidemiological analysis of bat rabies viruses in Brazil. J Vet Med Sci. 2005;67:647–52. PubMed http://dx.doi.org/10.1292/jvms.67.647

13. Kobayashi Y, Sato G, Kato M, Itou T, Cunha EM, Silva MV, et al. Genetic diversity of bat rabies viruses in Brazil. Arch Virol. 2007;152:1995–2004. PubMed http://dx.doi.org/10.1007/s00705-007-1033-y

14. Rohde RE, Mayes BC, Smith JS, Neill SU. Bat rabies, Texas, 1996–2000. Emerg Infect Dis. 2004;10:948–52. PubMed http://dx.doi.org/10.3201/eid1005.030719

15. Streicker DG, Turmelle AS, Vonhof MJ, Kuzmin IV, McCracken GF, Rupprecht CE. Host phylogeny constrains cross-species emergence and establishment of rabies virus in bats. Science. 2010;329:676–9. PubMed http://dx.doi.org/10.1126/science.1188836

16. Leslie MJ, Messenger S, Rohde RE, Smith J, Cheshier R, Hanlon C, et al. Bat-associated rabies virus in skunks. Emerg Infect Dis. 2006;12:1274–7. PubMed http://dx.doi.org/10.3201/eid1708.051526
